# Process-Oriented Measurement of Emotion Regulation: General and Specific Associations With Psychosocial Adjustment and Well-Being in (Pre-)Adolescence

**DOI:** 10.3389/fpsyt.2022.904389

**Published:** 2022-06-23

**Authors:** Jana-Elisa Rueth, Arnold Lohaus

**Affiliations:** Developmental Psychology and Developmental Psychopathology, Department of Psychology, Bielefeld University, Bielefeld, Germany

**Keywords:** emotion regulation, process model of emotion regulation, internalizing problems, externalizing problems, prosocial behavior, well-being, adolescence

## Abstract

The development of emotion regulation (ER) is associated with children’s and adolescents’ psychosocial adjustment and well-being. In this regard, previous studies have examined the role of different ER strategies, which can be characterized as being functional (e.g., reappraisal, problem solving) or dysfunctional (e.g., suppression, rumination). Based on the process model of emotion regulation, the strategies can also be classified according to their temporal position within the emotion generative process, with five families of ER strategies being proposed: situation selection, situation modification, attentional deployment, cognitive change, and response modulation. This study aimed to examine the role of ER for adolescents’ psychosocial adjustment (internalizing and externalizing problems, prosocial behavior) and well-being. First, associations were investigated on a more general level by distinguishing between functional and dysfunctional ER. Second, relations were examined on a more specific level by additionally distinguishing between the five families of ER strategies as suggested in the process model of ER. Questionnaire self-reports of *N* = 1,727 German children and adolescents (55% girls) aged 9–18 years (*M* = 13.03, *SD* = 1.75) collected in schools were analyzed. Path analyses showed that more functional and less dysfunctional ER in general is associated with fewer internalizing and externalizing symptoms, and higher well-being. Prosocial behavior was only positively related to functional but not dysfunctional ER. Analyses of associations on the level of specific categories of ER strategies generally showed a similar pattern, but in part indicated differential associations with the dependent variables: Internalizing problems were particularly associated with functional situation selection, dysfunctional cognitive change, and dysfunctional response modulation. Externalizing problems were associated with functional situation selection and response modulation, as well as numerous dysfunctional strategies, none of which were particularly salient. Similarly, numerous rather than single specific associations emerged between prosocial behavior and the five categories of functional ER strategies. Well-being was particularly associated with functional situation selection and dysfunctional cognitive change. Overall, a more precise assessment of ER, as implemented in this study, could not only advance research in this field, but can also be helpful in planning and evaluating prevention and intervention programs.

## Introduction

Emotions play an important role in everyday life and the appropriate processing, expression, and regulation of emotions is a crucial developmental task in childhood and adolescence. Empirical findings suggest that emotion regulation (ER) is not only associated with healthy development and well-being but also with psychopathological symptoms (1–4). ER can be defined as “the processes by which individuals influence which emotions they have, when they have them, and how they experience and express these emotions” (p. 275) (5). The regulatory processes that emerge in emotion-triggering situations may be conscious or unconscious, automatic or controlled, and include specific strategies that individuals use to accomplish their regulatory goal (5, 6).

According to the hedonistic principle, individuals typically seek to maximize pleasant and minimize (especially permanent) unpleasant feelings (7). In line with this assumption, it can be assumed that successfully reducing negative emotions (e.g., trying to calm down when being angry) and increasing positive emotions (e.g., sharing good news with close friends) is associated with higher well-being and fewer psychopathological symptoms. Hence, strategies serving these goals (e.g., reappraisal, problem solving) can be described as functional or adaptive (e.g., 1, 8, 9). If individuals fail to achieve the regulatory goal or if negative emotions are increased and positive emotions are decreased too frequently (e.g., when suppressing emotions or dwelling on problems), this is most likely associated with lower well-being and more behavioral problems. Accordingly, these strategies (e.g., rumination, suppression, catastrophizing) can be described as dysfunctional or maladaptive (e.g., 1, 8–12).

In addition to distinguishing between different regulatory goals, the ER strategies used by individuals can be organized along the time course of the emotion generative process. The *Process Model of Emotion Regulation* (5, 6) provides a widely used theoretical framework in this regard. It is based on the *Modal Model of Emotion* (13), which describes four distinguishable phases of the emotion generative process: First, the *situation* an individual faces, which can be internal (e.g., thoughts and beliefs) or external (e.g., separation from a caregiver). Second, the *attention* that is paid to certain aspects of the situation (e.g., frightening noises when being all alone in the dark). Third, *appraisal* processes during the evaluation of a situation (e.g., as threatening or challenging). Fourth, the person’s *response* to the emotion that has emerged (e.g., crying in sadness or fear).

According to the process model, regulatory efforts build upon these four phases of the emotion generative process. However, as regulation can already begin before an individual enters a potentially emotion-evoking event (i.e., the situation), five (and not only four) families of ER strategies are distinguished here (5): *Situation selection* (e.g., avoidance, seeking situations with positive emotional valence), *situation modification* (e.g., problem solving, seeking social support), *attentional deployment* (e.g., rumination, distraction), *cognitive change* (e.g., reappraisal, acceptance, catastrophizing), and *response modulation* (e.g., expressive suppression, aggressive reactions, physical exercising, relaxation). The latter of these five categories comprises strategies that are employed when the emotion is already fully present and has therefore been labeled as response-focused ER. In contrast, strategies that are used proactively and earlier in the emotion generative process make the experience of an emotion more or less likely and have therefore been described as antecedent-focused ER (5, 14).

The process-oriented approach provides a strong theoretical foundation for research in this area, as regulatory efforts can be systematically assessed and examined according to the five families of ER strategies (15–17). In the long term, this might provide a better understanding of causes and consequences of ER as well as the underlying mechanisms (13), also with regard to its role for the development of children and adolescents. Early and middle adolescence is characterized by a decrease in subjective well-being (18) and an increase in the likelihood of developing mental health problems (19). This might be due to the multiple challenges that youth face in this life stage, such as psychological (e.g., increasing cognitive abilities), biological (e.g., onset of puberty, brain maturation), and social changes (e.g., detachment from parents, changes in peer relationships) (e.g., 20, 21). The availability and use of appropriate ER strategies is an important prerequisite to cope with these challenges. Thus, the following sections elaborate on the associations of ER (in general and with regard to specific strategies) with well-being and psychosocial adjustment, namely internalizing and externalizing problems as well as prosocial behavior.

### Associations of Emotion Regulation With Psychosocial Adjustment

Based on the taxonomy of Achenbach (22), research in this field often distinguishes between internalizing and externalizing symptoms as two negative types of psychosocial adjustment (or maladjustment). The internalizing dimension includes problems within the person, such as depression, anxiety, social withdrawal, or somatic problems (e.g., headaches), while the externalizing dimension comprises conflicts with social interaction partners and the environment, such as conduct problems, hyperactivity, aggression, destructive behavior, or delinquency. Overall, findings of numerous cross-sectional and longitudinal studies, meta-analyses, and literature reviews suggest that more functional and less dysfunctional ER are associated with fewer (non-clinical) problem behaviors and (clinically relevant) psychopathological disorders across different age groups (e.g., 1–3, 9, 23). ER has even been proposed as a transdiagnostic factor for different (internalizing and externalizing) symptoms (e.g., 24, 25). In general, associations appear to be higher for dysfunctional ER strategies (1, 26, 27), suggesting that the presence of dysfunctional strategies is more important for emotional and behavior problems than the absence of functional strategies.

With regard to the role of specific ER strategies for internalizing and externalizing symptoms, a large number of studies has focused on two ER strategies in particular: Cognitive reappraisal, which is a typical antecedent-focused strategy representing the category of functional cognitive change, and suppression, which is a typical response-focused strategy representing the category of dysfunctional response modulation (8, 28). Studies with young adults (e.g., 8, 28–30) or adolescents (27, 31, 32) suggest that a more frequent use of reappraisal is associated with fewer depressive symptoms, whereas more suppression is associated with more symptoms of depression, social anxiety, and more externalizing problem behavior. However, meta-analyses (1, 23, 30) showed only small to medium effect sizes for the negative relation of cognitive reappraisal and the positive relation of suppression with negative indicators of mental health. In addition to these two ER strategies, there is a considerable body of research on rumination, which belongs to the category of dysfunctional attentional deployment. It has been found to be positively associated with a number of different psychopathological symptoms such as depression, anxiety, eating disorders, and substance abuse (25, 33, 34). In meta-analyses, large positive effect sizes for associations of rumination with anxiety and depressive symptoms were found (1, 23). Functional ER strategies that were included in empirical studies are problem solving (as functional situation modification), and acceptance (as functional cognitive change), which showed medium-sized negative associations especially with internalizing problems (1, 23).

Very few studies examined several different ER strategies simultaneously as predictors of internalizing and externalizing symptoms. Garnefski et al. (34–36) addressed this research gap, but focused solely on cognitive strategies: More frequent dysfunctional rumination (as attentional deployment), catastrophizing (as cognitive change), and self-blame (as response modulation), and less frequent functional reappraisal (as cognitive change) were associated with more internalizing symptoms (i.e., depression and anxiety). Externalizing problems, on the other hand, were only associated with higher scores on positive refocusing (i.e., thinking of pleasant things and experiences) as one type of functional attentional deployment. For several other strategies (e.g., acceptance, blaming others), however, no significant associations were found. These findings emphasize that different ER strategies may vary in their importance for different qualities of problem behavior as indicators of adolescents’ psychosocial adjustment, and that they should be examined more systematically in a theory-based manner.

Beyond the well-studied association of ER with emotional and behavioral problems, its importance for positive indicators of psychosocial adjustment has also been emphasized (37). In this regard, prosocial behavior (i.e., sharing, comforting, helping), which can be described as “voluntary behavior intended to benefit another” (p. 646) (38), constitutes one important aspect. In general, well-regulated individuals (i.e., using more functional and fewer dysfunctional ER strategies) are assumed to be more likely to show prosocial and empathy-related behaviors. In contrast, distressed and dysregulated individuals might be too self-focused in social interactions to exhibit prosocial behavior (39). Empirical studies with children and adolescents found that better ER-skills are positively associated with more prosocial behavior and empathy (40–44). Studies looking at associations of prosocial behavior with specific ER strategies suggest that a more frequent use of reappraisal (as functional cognitive change) and a less frequent use of suppression (as dysfunctional response modulation) is associated with more prosocial behavior, empathy and helping behavior in adolescence and adulthood (45, 46).

### Associations of Emotion Regulation With Well-Being

Given that health “is a state of complete physical, mental and social well-being and not merely the absence of disease or infirmity” (p. 1) as defined by the World Health Organization (47), the well-being of youth represents another important variable in the context of ER research. Subjective well-being has been defined as individuals’ evaluation of various aspects of life, such as general life satisfaction and domain-specific satisfaction (e.g., related to health, family, leisure), as well as their emotional experiences of pleasant and unpleasant affect (48, 49). In general, being able to deal with emotionally challenging situations adequately—which includes the use of appropriate ER strategies—seems to be important for individuals’ well-being and life satisfaction (50, 51).

Empirical studies on associations with specific ER strategies again focused mainly on cognitive reappraisal and expressive suppression. Most of these studies were conducted with young adults and found that—in general—a more frequent use of reappraisal and a less frequent use of suppression is associated with higher self-reported well-being and life-satisfaction (8, 29). A meta-analysis (30) showed small effect sizes for the association of cognitive reappraisal and expressive suppression with positive indicators of mental health (e.g., well-being, life-satisfaction, positive affect). However, less than a quarter of participants included in this meta-analysis were from studies with children and adolescents. Two studies that specifically focused on adolescents also found positive associations for reappraisal and negative associations for suppression with well-being (4, 46). One of the few studies including a wider range of ER strategies used by adult participants (10) found that reappraisal and refocus on planning (as functional situation modification) are positively related to well-being, while rumination (as dysfunctional attentional deployment), catastrophizing, and self-blame (both as dysfunctional cognitive change) showed negative associations. However, there were also some ER strategies (e.g., acceptance, positive refocusing) that did not show meaningful associations, which suggests that different ER strategies may vary in their effectiveness in promoting individuals’ well-being.

### Hypotheses and Research Questions

There has been a considerable amount of research on the role of functional and dysfunctional ER for internalizing and externalizing problems. Some studies also examined associations with prosocial behavior and well-being. In addition to empirical investigations of these general associations with functional and dysfunctional ER, researchers have described the relevance of specific strategies out of the five families of ER strategies for psychopathology and well-being (e.g., cognitive reappraisal, expressive suppression, and rumination). However, to the knowledge of the authors, no study so far has investigated the role of all families of ER strategies simultaneously for different negative and positive indicators of psychosocial adjustment and well-being.

Hence, the aim of this study was to examine general (Research Question 1) as well as specific associations (Research Question 2) of ER with well-being and psychosocial adjustment in adolescence. It was expected that (1a) more functional and (1b) less dysfunctional ER is associated with fewer internalizing and externalizing problems, more prosocial behavior, and higher well-being. Additionally, specific associations between the five families of (2a) functional and (2b) dysfunctional ER strategies (situation selection, situation modification, attentional deployment, cognitive change, and response modulation) and internalizing problems, externalizing problems, prosocial behavior, and well-being were exploratively investigated.

## Materials and Methods

### Sample and Procedure

Data were collected between December 2019 and December 2021 in northwestern Germany (in the regions of North Rhine-Westphalia and Lower Saxony) after all procedures had been approved by the ethics committee of Bielefeld University. Children and adolescents were recruited via schools, which were located in both urban and rural regions. Participation was voluntary, but only possible if informed consent signed by their parents was provided. The questionnaires were completed during a 45-min school lesson in the presence of at least one trained instructor who guided the class through the survey and answered questions. All items were read to younger participants (5–7th grade) to compensate for reading difficulties. Self-reports of *N* = 1,727 children and adolescents (55% female) were included in this study.^1^ Participants were 9–18 years old (*M* = 13.03, *SD* = 1.75) and attended grades 5–10 of grammar (41%), comprehensive (12%), or intermediate secondary schools (47%). The Total Difficulties Score of the Strengths and Difficulties Questionnaire [SDQ; (52, 53)] showed that 14% of the sample had increased values with sum scores ≥20, which is slightly higher compared to previous studies (e.g., 53).

### Measures

#### Emotion Regulation

The newly developed Process-Oriented Emotion Regulation Measure for Children and Adolescents [POEM-CA; (54)] was used to assess children’s and adolescents’ ER. Participants indicated on a 4-point scale (1 = *not true at all*, 2 = *rather not true*, 3 = *rather true*, and 4 = *totally true*) which strategies they use to regulate their emotions. Children’s and adolescents’ ER strategy use is measured with five functional and five dysfunctional primary subscales, namely Situation Selection, Situation Modification, Attentional Deployment, Cognitive Change, and Response Modulation. Each of these 10 subscales consists of 6 items, except for the functional and dysfunctional Attentional Deployment scales with 5 items each. Thus, the POEM-CA comprises a total of 58 items, which can also be combined to form secondary subscales for functional and dysfunctional ER (with 29 items each). Internal consistencies (Cronbach’s α) were excellent for the secondary subscales (α_functional_ = 0.92; α_dysfunctional_ = 0.91) and acceptable for the primary subscales (0.68 ≤ α ≤ 0.83). All coefficients, item examples, the respective number of items, means and standard deviations of the primary and secondary subscales are shown in Table 1. The POEM-CA previously showed satisfactory construct and criterion-related validity (54): Second-order confirmatory factor analyses showed acceptable fit indices for the two measurement models of functional and dysfunctional ER. Furthermore, medium to high correlations with other measures of ER were found. For example, functional ER was significantly associated with Reappraisal [*r* = 0.74, *p* < 0.001; measured by the Regulation of Emotions Questionnaire for Children and Adolescents; ERQ-CA; (31, 55)] and adaptive ER [*r* = 0.80, *p* < 0.001; measured by the Questionnaire for the Measurement of ER in Children and Adolescents; FEEL-KJ; (56)]. Dysfunctional ER showed meaningful associations with Suppression (*r* = 0.49, *p* < 0.001; ERQ-CA) and maladaptive ER (*r* = 0.73, *p* < 0.001; FEEL-KJ).

**TABLE 1:** Item examples, number of items, and internal consistencies (Cronbach’s α) of the POEM-CA primary and secondary subscales.

Subscale	Item example	No. of items	α
Functional ER		29	0.92
(F1) Situation selection	I do things that put me in a good mood.	6	0.77
(F2) Situation modification	When I am scared of something, I ask someone for help.	6	0.71
(F3) Attentional deployment	When I am sad, I try to think of something nice.	5	0.83
(F4) Cognitive change	When I get angry, I think about what I can learn from the situation.	6	0.75
(F5) Response modulation	When I’m agitated or nervous, I do something to relax.	6	0.72
Dysfunctional ER		29	0.91
(D1) Situation selection	I do things even though I know I’ll be angry about it afterward.	6	0.79
(D2) Situation modification	When I am feeling down, I don’t see any way to improve the situation.	6	0.79
(D3) Attentional deployment	I can’t get rid of thoughts about something that scares me.	5	0.76
(D4) Cognitive change	When I feel sad, I think it’s just me feeling that way.	6	0.73
(D5) Response modulation	When someone annoys me, my emotions easily run wild.	6	0.68

*ER, emotion regulation. 1,553 ≤ N ≤ 1,683.*

#### Psychosocial Adjustment

Children’s and adolescents’ psychosocial adjustment was measured with the German self-report version of the Strengths and Difficulties Questionnaire [SDQ; (52, 53)]. The 25 items were answered on a 3-point scale (1 = *not true*, 2 = *somewhat true*, and 3 = *certainly true*) to assess (A) Emotional Problems, (B) Peer Relationship Problems, (C) Conduct Problems, (D) Hyperactivity/Inattention, and (E) Prosocial Behavior with five items each. As suggested by Goodman (57), the four problem-focused primary subscales were combined to the broader secondary scales Internalizing Problems (comprising subscales A and B) and Externalizing Problems (comprising subscales C and D). The mean for each participant was calculated if no more than 30% of answers were missing. Internal consistencies were satisfactory (α_internalizing_ = 0.74; α_externalizing_ = 0.73, α_prosocial_ = 0.69) and in line with previous studies (53, 57, 58).

#### Well-Being

The KIDSCREEN-10 (59, 60) was used to measure children’s and adolescents’ well-being. The 10 self-report items were answered on a 5-point scale (1 = *never/not at all*, 2 = *seldom/slightly*, 3 = *quite often/moderately*, 4 = *very often/very*, and 5 = *always/extremely*) and combined to a total score by averaging the items if no more than three answers were missing. The internal consistency was good (α = 0.84) and comparable to previous studies (e.g., 59).

### Statistical Analyses

IBM SPSS Version 28 was used to compute the scale means for each participant and for all preliminary analyses (i.e., internal consistencies, sample means, and inter-correlations). Path models were calculated in Mplus Version 8.5 (61) and goodness of fit was evaluated using the indices and related cut-offs suggested by Hu and Bentler (62): A comparative fit index (CFI) ≥ 0.95, a root mean square error of approximation (RMSEA) ≤ 0.06, and a standardized root mean square residual (SRMR) ≤ 0.08 indicate a good model fit. Although deviations from normal distribution were only minor, the robust maximum likelihood estimator (MLR) was used for parameter estimation. Some multivariate outliers were found, but since these were not due to data entry errors and the calculations with and without these outliers did not yield meaningful differences in the results, they remained in the sample. The percentage of missing values was low for the POEM-CA primary and secondary subscales (< 1%) and the SDQ subscales (< 2%), but slightly higher for the KIDSCREEN total score (< 10%), because this questionnaire was the last of the survey and could not be completed in some classes due to time constraints. All missing values were handled using Full-Information Maximum Likelihood (FIML) estimation.

The research questions of this study were examined by computing two path models with manifest variables. In both models, the SDQ subscales (internalizing and externalizing problems, prosocial behavior) and the KIDSCREEN total score (well-being) were entered simultaneously as dependent variables. To examine general associations of these variables with functional and dysfunctional ER (Research Question 1), the outcome variables were regressed on the two secondary subscales of the POEM-CA as correlated independent variables (Model 1). To examine more specific associations (Research Question 2), the outcome variables were regressed on the 10 primary subscales of the POEM-CA as correlated independent variables (Model 2). In both models, correlations between the residuals of the dependent variables were allowed for the KIDSCREEN total score with all SDQ-subscales and for externalizing problems with prosocial behavior. As preliminary analyses revealed significant associations of age and sex with the variables of interest (see Table 2), they were included in both models as predictors of the dependent variables and correlated with all independent variables.

**TABLE 2:** Inter-correlations, means, and standard deviations of study variables: emotion regulation, psychosocial adjustment, well-being, age, and sex.

	F	F1	F2	F3	F4	F5	D	D1	D2	D3	D4	D5	INT	EXT	PRO	Age	Sex^a^	*M* (*SD*)
F	−															*−0.02*	*−0.05*	2.62 (0.50)
F1	0.78	−														*−0.04*	*0.04*	2.95 (0.56)
F2	0.80	0.56	−													*0.00*	–0.13	2.53 (0.58)
F3	0.80	0.51	0.52	−												–0.11	*−0.02*	2.52 (0.74)
F4	0.80	0.48	0.55	0.56	−											*0.04*	*−0.03*	2.47 (0.65)
F5	0.86	0.63	0.61	0.62	0.61	−										*0.03*	*−0.04*	2.63 (0.58)
D	–0.40	–0.42	–0.29	–0.30	–0.30	–0.33	−									*0.03*	–0.26	2.46 (0.51)
D1	–0.31	–0.41	–0.20	–0.27	–0.18	–0.23	0.74	−								0.12	–0.12	2.24 (0.65)
D2	–0.53	–0.46	–0.52	–0.40	–0.35	–0.40	0.77	0.49	−							0.13	–0.11	2.42 (0.67)
D3	–0.19	–0.21	*−0.04*	–0.15	–0.18	–0.19	0.76	0.43	0.37	−						–0.10	–0.27	2.65 (0.68)
D4	–0.26	–0.29	–0.14	–0.15	–0.26	–0.23	0.81	0.47	0.49	0.64	−					*−0.05*	–0.28	2.39 (0.67)
D5	–0.27	–0.26	–0.22	–0.21	–0.20	–0.22	0.82	0.46	0.59	0.59	0.58	−				*0.01*	–0.22	2.65 (0.60)
INT	–0.29	–0.36	–0.18	–0.22	–0.21	–0.22	0.65	0.45	0.47	0.50	0.56	0.55	−			*0.05*	–0.29	1.67 (0.38)
EXT	–0.35	–0.33	–0.24	–0.23	–0.26	–0.34	0.42	0.33	0.30	0.33	0.33	0.33	0.31	−		–0.17	*0.06*	1.67 (0.36)
PRO	0.38	0.29	0.36	0.26	0.31	0.33	*−0.08*	–0.12	–0.18	*0.05*	*−0.03*	*−0.01*	*−0.04*	–0.32	−	0.09	–0.20	2.57 (0.39)
WB	0.42	0.44	0.32	0.29	0.31	0.31	–0.55	–0.39	–0.47	–0.37	–0.49	–0.43	–0.63	–0.33	0.19	*−0.08*	0.25	3.76 (0.70)

*1,547 ≤ N ≤ 1,722. ^a^girls = 0, boys = 1. F, Functional Emotion Regulation; F1, Functional Situation Selection; F2, Functional Situation Modification; F3, Functional Attentional Deployment; F4, Functional Cognitive Change; F5, Functional Response Modulation; D, Dysfunctional Emotion Regulation; D1, Dysfunctional Situation Selection; D2, Dysfunctional Situation Modification; D3, Dysfunctional Attentional Deployment; D4, Dysfunctional Cognitive Change; D5, Dysfunctional Response Modulation; INT, Internalizing Problems; EXT, Externalizing Problems; PRO, Prosocial Behavior; WB, Well-being. Correlation coefficients were significant at p < 0.001, except for correlations in gray italics.*

Additionally, to investigate whether the specific regression paths in Model 2 differed significantly from one another, pairwise parameter comparisons were conducted. These comparisons were only performed for significant paths and within the combination of one dependent variable and one set of strategy families (separated by functional and dysfunctional ER). For example, the path from dysfunctional response modulation to internalizing problems was compared with each of the four other paths of dysfunctional ER (situation selection, situation modification, attentional deployment, and cognitive change) with internalizing problems. For this purpose, equality constraints were added to the model for the respective parameters, and the nested models were compared using the difference test for scaled χ^2^-values (63). A significant model comparison indicates a significant difference between the two paths that were compared.

## Results

### Preliminary Analyses

Means, standard deviations and inter-correlations of all measures of ER, psychosocial adjustment, and well-being as well as correlations with age and sex are presented in Table 2. The associations were mostly significant and in expected directions. Prosocial behavior showed only small and in part non-significant associations with dysfunctional ER and internalizing problems. Associations with age and sex suggested that younger participants showed less externalizing problems (*r* = −0.17) than older participants, and girls (in comparison to boys) showed more dysfunctional ER (*r* = −0.26), internalizing problems (*r* = −0.29), and prosocial behavior (*r* = −0.20), and reported lower well-being (*r* = 0.25; all significant at *p* < 0.001).

### General Associations With Psychosocial Adjustment and Well-Being (Model 1)

**FIGURE 1:**
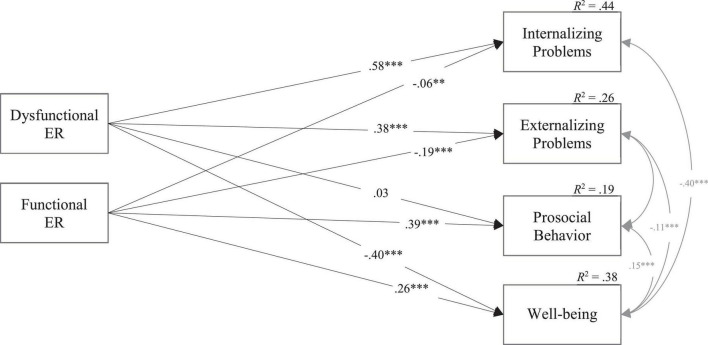
General associations of emotion regulation with psychosocial adjustment and well-being (Model 1). This path model shows general associations of adolescents’ functional and dysfunctional emotion regulation, measured by the secondary subscales of the POEM-CA, with their psychosocial adjustment (SDQ) and well-being (KIDSCREEN). For reasons of simplification, associations with age and sex (covariates), and inter-correlations of the independent variables are not displayed. Statistics are standardized correlation and regression coefficients. *N* = 1,727. χ^2^ = 12.220, *df* = 2, CFI = 0.996, RMSEA = 0.054, SRMR = 0.009. ^**^p < 0.01; ^***^p < 0.001.

The path model showed acceptable fit indizes, χ^2^ = 12.220, *df* = 2, CFI = 0.996, RMSEA = 0.054, SRMR = 0.009. The explained variance of the dependent variables was highest for internalizing problems (*R*^2^ = 0.44) and well-being (*R*^2^ = 0.38), but also significant for externalizing problems (*R*^2^ = 0.26) and prosocial behavior (*R*^2^ = 0.19). All but one of the path coefficients were significant and in expected directions (see Figure 1 and Supplementary Table 1).

More dysfunctional ER was significantly associated with more internalizing (β = 0.58, *p* < 0.001) and externalizing symptoms (β = 0.38, *p* < 0.001), and lower well-being (β = −0.40, *p* < 0.001). No significant relation between dysfunctional ER and prosocial behavior was found (β = 0.03, *p* = 0.182). In addition, more frequent functional ER was significantly associated with less internalizing (β = −0.06, *p* = 0.003) and externalizing problems (β = −0.19, *p* < 0.001), and more prosocial behavior (β = 0.39, *p* < 0.001) and well-being (β = 0.26, *p* < 0.001). Overall, associations between dysfunctional ER and problem behavior and well-being were strongest, but functional ER was also associated with all outcome measures and was the best predictor of prosocial behavior. Age and sex (girls = 0, boys = 1) as covariates both showed significant associations (*p* < 0.001) with externalizing problems (β_age_ = −0.18, β_sex_ = 0.14), prosocial behavior (β_age_ = 0.10, β_sex_ = −0.17), and well-being (β_age_ = −0.07, β_sex_ = 0.17). However, only sex (β_sex_ = −0.14), but not age, was significantly associated with internalizing problems.

### Specific Associations With Psychosocial Adjustment and Well-Being (Model 2)

**FIGURE 2:**
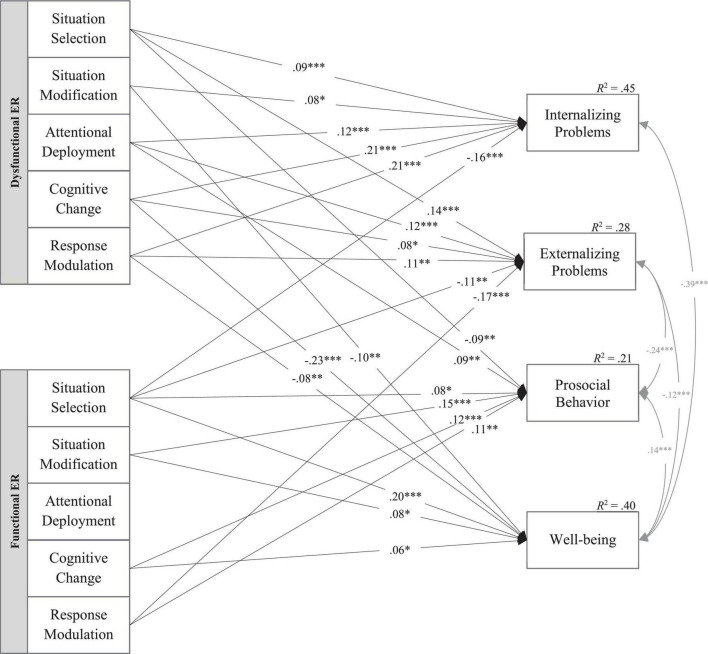
Specific associations of emotion regulation with psychosocial adjustment and well-being (Model 2). This path model shows specific associations of adolescents’ functional and dysfunctional ER, measured by the primary subscales of the POEM-CA, with their psychosocial adjustment (SDQ) and well-being (KIDSCREEN). For reasons of simplification, non-significant paths, associations with age and sex (covariates), and inter-correlations of the independent variables are not displayed. *N* = 1,727. χ^2^ = 9.746, *df* = 2, CFI = 0.997, RMSEA = 0.047, SRMR = 0.004. *p < 0.05; ^**^p < 0.01; ^***^p < 0.001.

The path model fitted the data well, χ^2^ = 9.746, *df* = 2, CFI = 0.997, RMSEA = 0.047, SRMR = 0.004. Similar to Model 1, the explained variance of the dependent variables in Model 2 was highest for internalizing problems (*R*^2^ = 0.45) and well-being (*R*^2^ = 0.40), but also significant for externalizing problems (*R*^2^ = 0.28) and prosocial behavior (*R*^2^ = 0.21). Numerous significant associations were found in the expected directions (see Figure 2 and Supplementary Table 2).

Internalizing problems were positively associated with all categories of dysfunctional ER. Dysfunctional cognitive change (β = 0.21, *p* < 0.001) and response modulation (β = 0.21, *p* < 0.001) showed the largest path coefficients, whereas dysfunctional attentional deployment (β = 0.12, *p* < 0.001), situation modification (β = 0.08, *p* = 0.010), and situation selection (β = 0.09, *p* < 0.001) showed rather small regression weights. Pairwise parameter comparisons revealed that the two largest regression weights (dysfunctional cognitive change and response modulation) were significantly higher than all other dysfunctional paths predicting internalizing problems (4.766 ≤ Δχ^2^ ≤ 10.191, Δ*df* = 1, *p* < 0.05). Regarding functional ER, only less situation selection (β = −0.16, *p* < 0.001) was significantly associated with more internalizing problems.

Externalizing problems were positively related to all but one category of dysfunctional ER: No significant association with situation modification was found (β = 0.01, *p* = 0.709), but more dysfunctional situation selection (β = 0.14, *p* < 0.001), attentional deployment (β = 0.12, *p* < 0.001), cognitive change (β = 0.08, *p* = 0.010), and response modulation (β = 0.11, *p* = 0.001) were associated with more externalizing symptoms. With regard to functional ER, situation selection (β = −0.11, *p* = 0.001) and response modulation (β = −0.17, *p* < 0.001) were negatively related to externalizing problems. Neither for functional nor for dysfunctional ER strategy families did the pairwise parameter comparisons of the significant paths reveal meaningful differences.

Prosocial behavior was negatively associated with dysfunctional situation selection (β = −0.09, *p* = 0.001). Contrary to expectations, however, a positive association between prosocial behavior and dysfunctional attentional deployment was found (β = 0.09, *p* = 0.004). These two paths were significantly different from each other (Δχ^2^ = 17.359, Δ*df* = 1, *p* < 0.001) but were of similar magnitude. Additionally, prosocial behavior was positively related to all but one category of functional ER: More situation selection (β = 0.08, *p* = 0.022), situation modification (β = 0.15, *p* < 0.001), cognitive change (β = 0.12, *p* < 0.001), and response modulation (β = 0.11, *p* = 0.002) were associated with more prosocial behavior.

Well-being was negatively related to three of the five families of dysfunctional ER strategies: Cognitive change showed the largest path coefficient (β = −0.23, *p* < 0.001), whereas situation modification (β = −0.10, *p* = 0.004), and response modulation (β = −0.08, *p* = 0.009) showed rather small associations. The regression weight of dysfunctional cognitive change was significantly higher compared to dysfunctional situation modification (Δχ^2^ = 5.937 Δ*df* = 1, *p* = 0.015) and response modulation (Δχ^2^ = 6.794, Δ*df* = 1, *p* = 0.009). Regarding functional ER, more situation selection was associated with higher well-being (β = 0.20, *p* < 0.001). This path was significantly higher compared to the two other significant paths of functional situation modification (β = 0.08, *p* = 0.012, Δχ^2^ = 7.284, Δ*df* = 1, *p* = 0.007) and cognitive change (β = 0.06, *p* = 0.039; Δχ^2^ = 13.430, Δ*df* = 1, *p* < 0.001).

The covariates age and sex (girls = 0, boys = 1) were both significantly associated with internalizing problems (β_age_ = 0.05, *p* = 0.019; β_sex_ = −0.12, *p* < 0.001), externalizing problems (β_age_ = −0.16, *p* < 0.001; β_sex_ = 0.15, *p* < 0.001), prosocial behavior (β_age_ = 0.11, *p* < 0.001; β_sex_ = −0.15; *p* < 0.001), and well-being (β_age_ = −0.08, *p* = 0.001; β_sex_ = 0.16, *p* < 0.001).

## Discussion

The aim of this study was to examine the role of adolescents’ ER for their psychosocial adjustment (i.e., internalizing and externalizing problems, prosocial behavior) and well-being. First, associations were investigated on a more general level, by distinguishing between the two broader dimensions of functional (e.g., reappraisal, problem solving) and dysfunctional ER (e.g., rumination, suppression). Second, a finer distinction was made within the two categories of functional and dysfunctional ER by additionally distinguishing between the five families of ER strategies, namely situation selection, situation modification, attentional deployment, cognitive change, and response modulation (5, 6).

### General and Specific Associations With Psychosocial Adjustment

Overall, the findings on general associations (Model 1, Research Question 1) supported our hypotheses: More functional ER was associated with better psychosocial adjustment (i.e., fewer internalizing and externalizing symptoms, and more prosocial behavior), while more dysfunctional ER was associated with more internalizing and externalizing problems. These results are in line with previous studies (1–3, 9, 23) and—given the significant relations of functional and dysfunctional ER strategies with both, internalizing and externalizing symptoms—underline the transdiagnostic nature of ER that has been suggested by several researchers (e.g., 24, 25). However, associations between dysfunctional ER and problem behavior were more pronounced for internalizing symptoms (in comparison to externalizing symptoms). Also, consistent with previous studies (e.g., 26, 27), associations of ER with negative aspects of psychosocial adjustment were higher for dysfunctional strategies (compared with functional strategies). This might suggest that adolescents’ dysfunctional ER is more important for mental health than a reduced availability of functional strategies. However, dysfunctional ER was not associated with adolescents’ prosocial behavior, which was instead related to functional ER. This finding extends previous research with elementary school children (40) or specifically on anger regulation (41), and underlines the importance of functional ER strategies above and beyond the relevance of general dysfunctional ER, particularly with regard to prosocial behavior.

Regarding the specific associations (Model 2, Research Question 2) of the five families of functional and dysfunctional ER strategies (situation selection, situation modification, attentional deployment, cognitive change, and response modulation) with psychosocial adjustment, numerous significant associations were found in the expected directions. Internalizing problems were positively related to all strategy families of dysfunctional ER, but associations were most pronounced (and significantly higher) for cognitive change and response modulation. Thus, strategies such as catastrophizing, self-blame, and resignation (as examples of dysfunctional cognitive change) as well as suppression, aggressive reactions, and withdrawal (as examples of dysfunctional response modulation) seem to be most important for problems from the internalizing spectrum (e.g., anxiety and depressive symptoms). This suggests that dysfunctional strategies that are applied relatively late in the emotion generative process (i.e., close to the point where the emotion arises and is fully present) are more harmful in comparison to strategies earlier in the process. In contrast, functional situation selection (e.g., seeking situations with positive emotional valence), which is applied very early in the process, appears to have the most protective effect. All other functional and dysfunctional strategy families only showed small or even non-significant associations with internalizing problems. These findings are surprising given that strategies representing functional cognitive change (e.g., reappraisal, acceptance), functional situation modification (e.g., problem solving), and dysfunctional attentional deployment (e.g., rumination) have been found to be associated with anxiety and depression in previous studies (1, 23, 33, 64), and are most often focused in interventions. However, the prominent role of dysfunctional cognitive change and response modulation (found in the present study) is in line with results from a meta-analysis (17), which also used the process model as the theoretical framework to examine the effectiveness of different ER strategies for changes in emotional states (e.g., after emotion induction in experimental settings). Small to medium-sized effects for cognitive change and response modulation, but no effect for attentional deployment were found. Even though the authors did not distinguish between functional and dysfunctional strategies in a way that maps onto the assessment in the present study, the meta-analytic findings and results of our study emphasize the need to examine the relative importance of different categories of ER strategies in a theory-based manner.

Externalizing problems were positively related to most dysfunctional strategies, except situation modification. However, as path coefficients were only small and pairwise parameter comparisons did not reveal meaningful differences between the four (significant) strategy families, it does not appear that specific phases of the emotion generative process have a particularly high impact. The overall negative association of dysfunctional ER and symptoms from the externalizing spectrum (e.g., aggression, hyperactivity) thus seems to result from regulatory deficits in almost all phases of the emotion generative process. This is in line with results from Brenning et al. (27), who found that general emotion dysregulation better predicted externalizing problems than the specific dysfunctional strategy of suppression. For functional ER, only situation selection (e.g., seeking situations with positive emotional valence, avoiding anger) and response modulation (e.g., physical exercising, relaxation) were significantly associated with fewer externalizing problems. These families of functional strategies represent behavioral rather than cognitive ER. Interestingly, one study that analyzed profiles of dysfunctional ER also found externalizing problems to be associated with a more behavioral regulation style, whereas a cognitive regulation style was more strongly related to internalizing problems (65). The findings of our study extend these results with regard to functional ER and suggest that this distinction may also be useful for future research. Overall, the specific associations of functional and dysfunctional ER with externalizing problems were small to medium-sized in magnitude in this study. This reflects conclusions of previous research that the relationship between ER and externalizing problems (in comparison to internalizing problems) is less pronounced (1).

Prosocial behavior as a positive indicator of psychosocial adjustment showed the expected negative relation to dysfunctional situation selection (i.e., putting oneself in situations that result in a bad mood). Surprisingly, more dysfunctional attentional deployment (i.e., rumination) was significantly associated with more (instead of less) prosocial behavior. Rumination is characterized by repetitive negative thinking about things (e.g., events, social situations, and own emotional states) that are difficult to control (33). When ruminating about their own emotions, adolescents might also be very attentive to the emotions of their social interaction partners. In this regard, previous studies found that higher attention to others’ emotions is associated with more worry and rumination (66), and also more empathy (67) and prosocial behavior (58). Thus, the awareness of others’ emotions might play an important role for the positive association between dysfunctional attentional deployment and prosocial behavior found in this study and should be subject of future research. Regarding specific functional ER, with the exception of attentional deployment, all categories were significantly related to prosocial behavior, and pairwise parameter comparisons did not reveal meaningful differences. This suggests that several different functional ER strategies are equally beneficial for socially competent behavior. The findings extend previous research that mainly focused on cognitive reappraisal and suppression (45, 46) and indicate that also strategies such as relaxation (functional response modulation) or seeking social support and problem solving (functional situation modification) are relevant for prosocial behavior of adolescents. These functional strategies could thus ensure that sufficient cognitive capacities are available to respond to the needs and the emotional state of other persons and manage social interactions successfully, which seems to be particularly relevant when one’s own trait emotionality is high (39, 68).

### General and Specific Associations With Well-Being

The results of this study suggest that adolescents’ use of functional and dysfunctional ER strategies is not only related to their psychosocial adjustment, but is also important for their well-being, which constitutes an important component of overall psychological health (28, 47). In line with our hypotheses on general associations (Model 1, Research Question 1) and previous research (30, 50), higher well-being was associated with more functional and less dysfunctional ER.

With regard to specific associations (Model 2, Research Question 2), dysfunctional situation modification, cognitive change, and response modulation were negatively associated with well-being, whereas functional situation selection, situation modification and cognitive change showed significant positive associations. Previous studies with adolescents (4, 46) have focused solely on cognitive reappraisal (functional cognitive change) and suppression (dysfunctional response modulation), but although significant associations with these families of ER strategies were also found in this study, the regression weights were rather small. Instead, the pairwise parameter comparisons indicate that dysfunctional cognitive change (compared to other dysfunctional strategies) and functional situation selection (compared to other functional strategies) are most relevant for adolescents’ well-being. Thus, when adolescents use (dysfunctional) catastrophizing or self-blaming strategies (dysfunctional cognitive change), this seems to be most harmful for their well-being. Balzarotti and colleagues (10) also found medium-sized associations of catastrophizing and self-blame with subjective well-being in adulthood. However, direct statistical comparisons with other dysfunctional strategies, as implemented in this study, were not conducted and studies with adolescents are not yet available. Among the functional strategies, intentionally seeking out situations that can elicit positive emotions and avoiding situations that can trigger anger, for example, seem to be particularly important. Functional situation selection requires the ability to predict which situations potentially evoke specific emotions. Therefore, a certain experience with one’s own emotions, especially an understanding of the causes of emotions, is advantageous to be able to use this strategy successfully. However, especially with regard to anxiety, this strategy should not be used too frequently and inflexible, as it can result in social interactions or leisure activities being permanently and excessively avoided. In this case, the long term costs would exceed the short-term benefits (12). In the ER measure used in this study, the focus of functional situation selection is actually on seeking out positive situations and only occasionally on avoiding anger or sadness. Thus, from this perspective, the use of this family of ER strategies seems to be beneficial for the well-being of adolescents.

### Practical Relevance

In general, it is assumed that processing emotion-triggering situations and understanding the causes of emotions as well as their quality (e.g., anger, fear) rather than suppressing or ignoring them is beneficial for a healthy development. Because if individuals do not deal with their emotions, a prolonged emotional arousal can occur that remains diffusely in the background. In this case, the emotion becomes detached from the situational circumstances and can no longer be adequately processed and managed (8, 12, 69, 70). This makes the awareness and regulation of emotions important skills, especially in emotionally demanding situations and phases of life. Adolescence is such a developmental period in which individuals experience numerous challenges that also include and affect emotions and their regulation (20, 21, 71, 72).

The findings of this study—particularly the numerous and at the same time specific associations of the five families of ER strategies with psychosocial adjustment and well-being—emphasize, on two different levels, that ER is a useful target in prevention and intervention settings. At the first level, adolescents’ use of functional and dysfunctional ER strategies is not only associated with specific psychopathologies in clinical samples, but also with various positive and negative indicators of psychological health in a non-clinical sample. This supports the proposed transdiagnostic role of ER (e.g., 24, 25) and expands knowledge about its importance for prosocial behavior and well-being. Beyond the findings of this study (in a non-clinical sample), Sloan et al. (73) systematically reviewed the role of ER as a transdiagnostic factor in treatment of different clinically relevant psychopathologies (i.e., anxiety, depression, substance use, eating pathology, or borderline personality disorder). They concluded that interventions for several disorders result in a decrease in dysfunctional ER and parallel reductions in symptomatology in most cases, which underlines the importance of ER in general as a treatment target. At the second level, the process-oriented approach implemented in this study revealed specific associations of the five categories of ER strategies, particularly for internalizing symptoms and well-being. Empirical findings on these specific relationships expand knowledge about ER, and an application of this approach in diagnostics, prevention and intervention may be useful. This could increase therapeutic success or enhance the protective effects of prevention programs to better support adolescents in a phase of reduced well-being (18) and of increased risk for mental health problems (19). Moreover, the process-oriented approach could be useful for future research that aims at gaining a deeper understanding of the concept of ER. Respective theoretical foundations and ideas for future research are outlined in the following section.

### Future Research

Gross and Jazaieri (11) proposed a conceptual framework for examining associations of emotions and ER with psychopathological symptoms. For emotions, problematic intensity, duration, frequency, and type were suggested as relevant components for future research. Regarding ER, emotional awareness, regulatory goals, and ER strategies were proposed. In this study, we mainly focused on the role of different strategies as one aspect of ER, either at a more general level (functional and dysfunctional) or at a more specific level, distinguishing between the five families of ER strategies as proposed in the process model (5, 6). Regulatory goals have also been incorporated within the ER assessment, but emotion awareness has not been included. The way individuals perceive and evaluate their own and others’ emotions (emotional awareness) has been found to be significantly associated with ER (58, 74), emotional information processing (75), empathy (67), and mental health (9, 70, 76). Furthermore, there are other dimensions of emotions (e.g., duration, intensity) that need attention in future research in this field (11).

With regard to the ER strategies examined in this study, future research should also consider the interplay between functional and dysfunctional strategies as Aldao and Nolen-Hoeksema (77) found that functional ER was associated with psychopathological symptoms only when the use of dysfunctional strategies was high. Furthermore, since emotional responses in the final phase of the process may change the (internal or external) situation and emotional experiences are highly dynamic and reciprocal, the process is more complex and less straightforward than assumed. It is proposed that feedback loops can be drawn between the response phase and the other phases of the modal model and associated strategies [for a detailed description, see Gross and Thompson (13)]. Therefore, future studies should examine these dynamic aspects in more detail, as it could make a difference whether the process was gone through only once or whether one or more feedback loops have already occurred and regulatory efforts have started later.

### Strengths and Limitations

The most notable strength of this study is that all phases of the emotion generative process as proposed in the process model (5, 6) were included in the assessment of ER. In contrast to previous research, which largely either used the broader dimensions of functional and dysfunctional ER or specifically assessed selected strategies (mostly cognitive reappraisal and expressive suppression), this offers new possibilities for analyzing specific relations between the five families of ER strategies (6) and the outcome variables of interest. It should also be mentioned that multiple indicators of mental health (i.e., internalizing problems, externalizing problems, prosocial behavior, and well-being) were considered in this study, which provides a more comprehensive picture of mental health and allows simultaneous (multivariate) investigation of associations. By investigating the general and specific associations of ER with psychosocial adjustment and well-being in early and middle adolescence, this study also focuses on a vulnerable age group and (with regard to mental health problems) an important developmental phase in which youth show high emotional reactivity (21) and are at a greater risk of developing psychopathological symptoms (19). Furthermore, analyses were based on a large sample and all subscales showed at least satisfactory internal consistencies. However, the values were lower for prosocial behavior (α = 0.69) and dysfunctional response modulation (α = 0.68), possibly due to the small number of items and the heterogeneity of the constructs. For example, the dysfunctional response modulation scale contains items on both aggressive reactions and emotion suppression, which may very well be answered in opposite directions and yet both represent the fifth phase of the process model.

Despite several strengths of this study, some limitations have to be mentioned. First, only cross-sectional data were used, which does not allow conclusions about causality and directionality. Second, as only self-reports were assessed, results might not be generalized to other perspectives. Third, cultural differences were not considered as only data from German adolescents were available. Fourth, findings are restricted to a non-clinical population and associations might not apply to clinical samples in the same way. For example, the strategy of avoidance (situation selection) is underrepresented in the ER measure used in this study (54). Avoidance (i.e., avoiding situations in which negative emotions may arise) is the most prominent example for strategies that—in the short term and if not used too frequently on specific emotions (e.g., anger)—can be functional. Thus, in non-clinical populations (as reported in this study) it might be functional to avoid situations that might cause anger (e.g., unnecessary disputes with peers) and similar items are included in the POEM-CA (54). However, for individuals showing symptoms of social anxiety or specific phobia, avoidance might be harmful (e.g., avoiding social interactions) and other studies have assessed this strategy as dysfunctional ER. Therefore, the results of this study (seeing specific types of avoidance as functional situation selection) may not match results of previous studies that have assessed dysfunctional types of avoidance. Finally, the SDQ was used to assess adolescents’ internalizing and externalizing problems. This measure is appropriate for screening purposes (78, 79), showing good sensitivity scores for several disorders [e.g., conduct disorders, hyperactivity, depression; (80)]. However, identification is poorer for some anxiety disorders [e.g., separation anxiety, specific phobias, panic disorders; (80)], suggesting that a more thorough measurement of symptoms on the internalizing spectrum might provide more accurate results.

## Conclusion

Despite the mentioned limitations, it can be concluded that the multiple and yet specific associations of the five families of functional and dysfunctional ER strategies with psychosocial adjustment and well-being in adolescence emphasize the importance of ER in general. Findings further suggest that different specific strategies may vary in their effectiveness in promoting individuals’ well-being, but also in maintaining mental health. In this regard, the theory-based, more specific, and yet broad measurement of ER implemented in this study may be valuable for future research and useful in the context of prevention and intervention.

## Data Availability Statement

The raw data supporting the conclusions of this manuscript will be made available by the first author to any qualified researcher upon request without undue reservation. Requests to access these datasets should be directed to J-ER, jana-elisa.rueth@uni-bielefeld.de.

## Ethics Statement

The studies involving human participants were reviewed and approved by Ethics Committee of Bielefeld University (EUB). Written informed consent to participate in this study was provided by the participants’ legal guardian/next of kin.

## Author Contributions

J-ER collected and analyzed the data, interpreted the results, and drafted and revised the manuscript. AL critically reviewed and revised the manuscript. Both authors designed the study, read, and approved the final manuscript.

## Conflict of Interest

J-ER and AL developed the Process-Oriented Emotion Regulation Measure for Children and Adolescents (POEM-CA).

## Publisher’s Note

All claims expressed in this article are solely those of the authors and do not necessarily represent those of their affiliated organizations, or those of the publisher, the editors and the reviewers. Any product that may be evaluated in this article, or claim that may be made by its manufacturer, is not guaranteed or endorsed by the publisher.

## Footnotes

1 Data stem from the norm sample of the Process-Oriented Emotion Regulation Measure for Children and Adolescents (POEM-CA). In comparison to the results published in Rüth and Lohaus (54), this study only includes the subsamples in which the relevant outcome measures (internalizing and externalizing problems, prosocial behavior, and well-being) were assessed.
