# Supplementary material for: Process-Oriented Measurement of Emotion Regulation: General and Specific Associations With Psychosocial Adjustment and Well-Being in (Pre-)Adolescence
Source: Front Psychiatry. 2022 Jun 23;13:904389. doi: 10.3389/fpsyt.2022.904389 (PMC9259935; doi:10.3389/fpsyt.2022.904389)
Supplement: Supplementary file 1 [file Table_1.PDF]

Supplementary Material

Table S1

Model 1: Unstandardized and Standardized Coefficients, Standard Errors, and Significance of Regression Paths

	INT				EXT				PRO				WB			
	B	*SE*	β	*p*	*B*	*SE*	β	*p*	*B*	*SE*	β	*p*	*B*	*SE*	β	*p*
D	.43	.02	.58	< .001	-.14	.02	-.19	< .001	.31	.02	.39	< .001	.36	.04	.26	< .001
F	-.05	.02	-.06	.003	.27	.02	.38	< .001	.03	.02	.03	.182	-.55	.04	-.40	< .001
age	.01	.00	.03	.072	-.04	.00	-.18	< .001	.02	.01	.10	< .001	-.03	.01	-.07	< .001
sex[a]	-.11	.01	-.14	< .001	.10	.02	.14	< .001	-.14	.02	-.17	< .001	.23	.03	.17	< .001

Notes. N = 1727. χ^2 = 12.220, df = 2, CFI = .996, RMSEA = .054, SRMR = .009. [a]girls = 0, boys = 1. F = Functional Emotion Regulation, D = Dysfunctional Emotion Regulation, INT = Internalizing Problems, EXT = Externalizing Problems, PRO = Prosocial Behavior, WB = Well-being.

Table S2

Model 2: Unstandardized and Standardized Coefficients, Standard Errors, and Significance of Regression Paths

	INT				EXT				PRO				WB			
	B	*SE*	β	*p*	*B*	*SE*	β	*p*	*B*	*SE*	β	*p*	*B*	*SE*	β	*p*
D1	.05	.01	.09	< .001	.08	.02	.14	< .001	-.06	.02	-.09	.001	-.05	.03	-.05	.079
D2	.04	.02	.08	.010	.01	.02	.01	.709	-.02	.02	-.03	.329	-.10	.04	-.10	.004
D3	.07	.02	.12	< .001	.07	.02	.12	< .001	.05	.02	.09	.004	-.03	.03	-.03	.345
D4	.12	.02	.21	< .001	.05	.02	.08	.010	.00	.02	.00	.937	-.24	.03	-.23	< .001
D5	.13	.02	.21	< .001	.06	.02	.11	.001	.04	.02	.06	.065	-.09	.04	-.08	.009
F1	-.11	.02	-.16	< .001	-.07	.02	-.11	.001	.05	.02	.08	.022	.25	.04	.20	< .001
F2	.01	.02	.02	.555	.01	.02	.02	.516	.10	.02	.15	< .001	.10	.04	.08	.012
F3	-.01	.01	-.02	.580	.00	.02	.01	.778	.00	.02	.00	.959	.01	.03	.01	.632
F4	-.01	.02	-.01	.689	-.01	.02	-.02	.610	.07	.02	.12	< .001	.06	.03	.06	.039
F5	.03	.02	.05	.135	-.11	.02	-.17	< .001	.07	.02	.11	.002	-.02	.04	-.02	.606
age	.01	.00	.05	.019	-.03	.00	-.16	< .001	.02	.01	.11	< .001	-.02	.01	-.08	.001
sex[a]	-.09	.02	-.12	< .001	.11	.02	.15	< .001	-.12	.02	-.15	< .001	.22	.03	.16	< .001

Notes. $N = 1727$. $\chi^2 = 12.220$, $df = 2$, CFI = .996, RMSEA = .054, SRMR = .009. [a]girls = 0, boys = 1. F1 = Functional Situation Selection, F2 = Functional Situation Modification, F3 = Functional Attentional Deployment, F4 = Functional Cognitive Change, F5 = Functional Response Modulation, D1 = Dysfunctional Situation Selection, D2 = Dysfunctional Situation Modification, D3 = Dysfunctional Attentional Deployment, D4 = Dysfunctional Cognitive Change, D5 = Dysfunctional Response Modulation, INT = Internalizing Problems, EXT = Externalizing Problems, PRO = Prosocial Behavior, WB = Well-being.
